# A panel of blood-derived miRNAs with a stable expression pattern as a potential pan-cancer detection signature

**DOI:** 10.3389/fmolb.2022.1030749

**Published:** 2022-12-15

**Authors:** Amir Sabbaghian, Veronika Mussack, Benedikt Kirchner, Maria L. U. Bui, Mohammad Reza Kalani, Michael W. Pfaffl, Masoud Golalipour

**Affiliations:** ^1^ Department of Molecular Medicine, Advanced Technologies Faculty, Golestan University of Medical Science, Gorgan, Iran; ^2^ Department of Animal Physiology and Immunology, TUM School of Life Sciences, Technical University of Munich, Munich, Germany; ^3^ Cellular and Molecular Research Center, Golestan University of Medical Science, Gorgan, Iran

**Keywords:** miRNA, biomarker, cancer, small RNA-seq, stable gene expression

## Abstract

**Introduction:** MicroRNAs have a significant role in the regulation of the transcriptome. Several miRNAs have been proposed as potential biomarkers in different malignancies. However, contradictory results have been reported on the capability of miRNA biomarkers in cancer detection. The human biological clock involves molecular mechanisms that regulate several genes over time. Therefore, the sampling time becomes one of the significant factors in gene expression studies.

**Method:** In the present study, we have tried to find miRNAs with minimum fluctuation in expression levels at different time points that could be more accurate candidates as diagnostic biomarkers. The small RNA-seq raw data of ten healthy individuals across nine-time points were analyzed to identify miRNAs with stable expression.

**Results:** We have found five oscillation patterns. The stable miRNAs were investigated in 779 small-RNA-seq datasets of eleven cancer types. All miRNAs with the highest differential expression were selected for further analysis. The selected miRNAs were explored for functional pathways. The predominantly enriched pathways were miRNA in cancer and the P53-signaling pathway. Finally, we have found seven miRNAs, including miR-142-3p, miR-199a-5p, miR-223-5p, let-7d-5p, miR-148b-3p, miR-340-5p, and miR-421. These miRNAs showed minimum fluctuation in healthy blood and were dysregulated in the blood of eleven cancer types.

**Conclusion:** We have found a signature of seven stable miRNAs which dysregulate in several cancer types and may serve as potential pan-cancer biomarkers.

## Introduction

The discovery of microRNAs (miRNA) as the new player of the transcriptome has changed the field of molecular biology. miRNAs are single-stranded small non-coding RNAs composed of 18–22 nucleotides (Sarshar et al., 2020). The binding of miRNA to the target genes, especially to the 3′ untranslated region (UTR), induces post-transcriptional gene regulation (Huntzinger and Izaurralde, 2011; Ipsaro and Joshua-Tor, 2015). A single miRNA could potentially target several mRNAs. Therefore, dysregulation of miRNAs profoundly affects the expression of numerous genes that may lead to human diseases such as cancer (Dharap et al., 2013; Peng and Croce, 2016; Zhang et al., 2018). Many studies have confirmed that miRNAs play an essential role in various cancer-associated biological processes such as apoptosis, proliferation, metabolism, invasion, differentiation, immune response, and metastasis (Kabekkodu et al., 2018; Abkhooie et al., 2021; O'Brien et al., 2018; He et al., 2019).

miRNAs have been found in various biological samples, including peripheral blood, which is readily obtainable in significant amounts (El-Mogy et al., 2018; Hermann et al., 2019). Studies have shown that miRNAs are the most significant regulating RNA components existing in peripheral blood and could be applied as biomarkers with high specificity and sensitivity for detecting cancers (Buschmann et al., 2016; Bhome et al., 2017; Carter et al., 2017; Dumache, 2017; Yan et al., 2017). However, the application of miRNAs as diagnostic biomarkers has some significant challenges. Several factors such as data preprocessing and optimization, interpersonal differences, the interaction of miRNAs with serum molecules, and the retention time of samples have been reported as the causes of controversies regarding the use of miRNAs as diagnostic biomarkers (Witwer, 2015; Monzo et al., 2017; Xu et al., 2017; Zhou et al., 2017; Grätz et al., 2022). The circadian clock system is based on a transcription-translation feedback loop (Jennifer et al., 2012). The circadian cycle organizes the regulation of the daily timing of transcriptome (including miRNA) (Du et al., 2014; Zhou et al., 2021). Therefore, in gene expression studies, the sampling time becomes one of the significant factors affecting the gene expression results. In this study, we tried to find miRNAs in the blood with minimum fluctuation at several time points. We proposed that these miRNAs could be a better candidate for cancer detection. We present here a panel of seven miRNAs with the slightest oscillation in healthy peripheral blood, which showed a significant dysregulation in 11 types of cancers.

## Materials and methods

### Patients and samples

We conducted a detailed search in the Gene Expression Omnibus (GEO) database to find appropriate small RNA sequencing raw data, using the keywords “small-RNA seq”, “whole blood derived”, and “blood-derived cancer” resulting in 779 patient raw data sets from 11 cancer types (Table 1 and Table 2). Our input criteria only include datasets that obtained blood. The RNA seq raw data for healthy individuals (*n* = 90) were obtained from the study of (Haberberger et al., 2018; Mussack et al., 2021), including whole blood samples of ten healthy male individuals at nine time-points (0 h, 3 h, 6 h, 24 h, 48 h, 72 h, Day7, Day30, and day60). A table of clinical information of 10 healthy individuals was provided in Supplementary Table S1 (Supplementary File S1).

**TABLE 1:** Cancer small RNA-seq raw datasets. Eleven different cancer datasets with their specific controls in nine different tissues. One time series dataset obtained from ten healthy individuals including 90 samples (Mussack et al., 2021). All the samples were obtained from blood in each cancer. All datasets are available in Gene Expression Omnibus (GEO) database (c). AML: acute myelogenous leukemia, CLL: chronic lymphocytic leukemia, ALL: acute lymphocytic leukemia.

Type of cancer	Healthy/Disease (N)	RNA-seq platform	References
Lung adenocarcinoma	4/6	GPL20795 HiSeq X ten	GSE151963 Wang et al. (2021)
			Wang X 2020
Colorectal cancer	192/92	GPL9052 Illumina Genome Analyzer	GSE71008 Yuan et al. (2016)
			Lin M 2019
Colon cancer	10/15	Illumina HiSeq 2500	PRJNA540919 Min et al. (2019)
			Zhang Sh 2019
Pancreatic cancer	24/21	GPL16791 Illumina HiSeq 2500	GSE109319 Kim et al. (2019)
			Kim K 2019
AML	9/10	GPL18573 Illumina NextSeq 500	GSE128079 Pandita et al. (2019)
			Pandita A 2019
CLL	5/25	GPL18573 Illumina NextSeq 500	GSE123436 Kaur et al. (2020)
			Kaur G 2020
Biliary tract cancer	24/10	GPL16791 Illumina HiSeq 2500	GSE109319 Kim et al. (2019)
			Kim K 2019
Gastric cancer	12/36	GPL11154 Illumina HiSeq 2000	GSE130654 Tang et al. (2020)
			Tang S 2020
Nasopharyngeal cancer	6/6	GPL16791 Illumina HiSeq 2500	GSE163867 Zheng et al. (2021)
			Zheng W 2021
ALL	36/150	Illumina Nextseq 500	GSE89978 Wallaert et al. (2017)
			Wallaert A 2017
Prostate cancer	50/36	GPL9052 Illumina Genome Analyzer	GSE71008 Yuan et al. (2016)
			Yuan T
Healthy individuals	90/0	GPL16791 Illumina HiSeq 2500	PRJEB38354 Mussack et al. (2021)
			Veronika M

**TABLE 2:** The foldchange of 37 selected DEMs in 11 cancer datasets compared to control in respective datasets. The miRNAs with expression fold change levels inside the threshold window were discarded. Seven DEMs with the minimum oscillation (MAD) in healthy controls showed the highest dysregulation in 11 cancers cancer datasets. All miRNAs indicated MAD score <0.2. AML: acute myeloid leukemia, CLL: chronic lymphoid leukemia. ALL: acute lymphoid leukemia. MAD: median absolute fold change.

miRNA	Threshold window		Expression changes in cancer (Log2FC) compared to healthy controls in each dataset										
			AML	Biliary	CLL	Colorectal	Early colon	Gastric	Lung adenocarcinoma	Nasopharyngeal	Pancreatic	Prostate	All
	Upper border	Lower border											
miR-142-3p	−0.622	0.510	−1.491	−0.840	0.519	−0.773	−0.782	2.796	−1.678	0.296	−0.687	−0.792	1.697
miR-199a-5p	−0.111	0.114	2.240	−2.165	−3.271	−0.899	−0.333	1.069	−2.151	2.906	−1.070	−0.679	0.432
miR-223-5p	−0.326	0.278	−1.924	2.470	−0.004	−0.386	−0.042	1.550	0.968	1.684	1.496	−0.333	3.024
let-7d-5p	−0.263	0.426	0.623	−0.435	−0.967	−0.676	0.559	1.473	−0.876	0.162	−0.222	−0.271	−0.623
miR-148b-3p	−0.097	0.112	0.005	−0.834	−1.409	−0.666	−0.233	-0.841	−1.400	1.248	−0.675	−0.135	0.592
miR-340-5p	−0.143	0.258	−2.075	1.286	0.533	0.169	−1.467	−0.769	−1.948	1.525	0.915	−0.080	2.611
miR-421	−0.354	0.317	−0.452	1.276	−1.172	0.368	−0.885	−1.709	−0.680	0.441	0.990	−0.083	−0.533

### Study design

**FIGURE 1:**
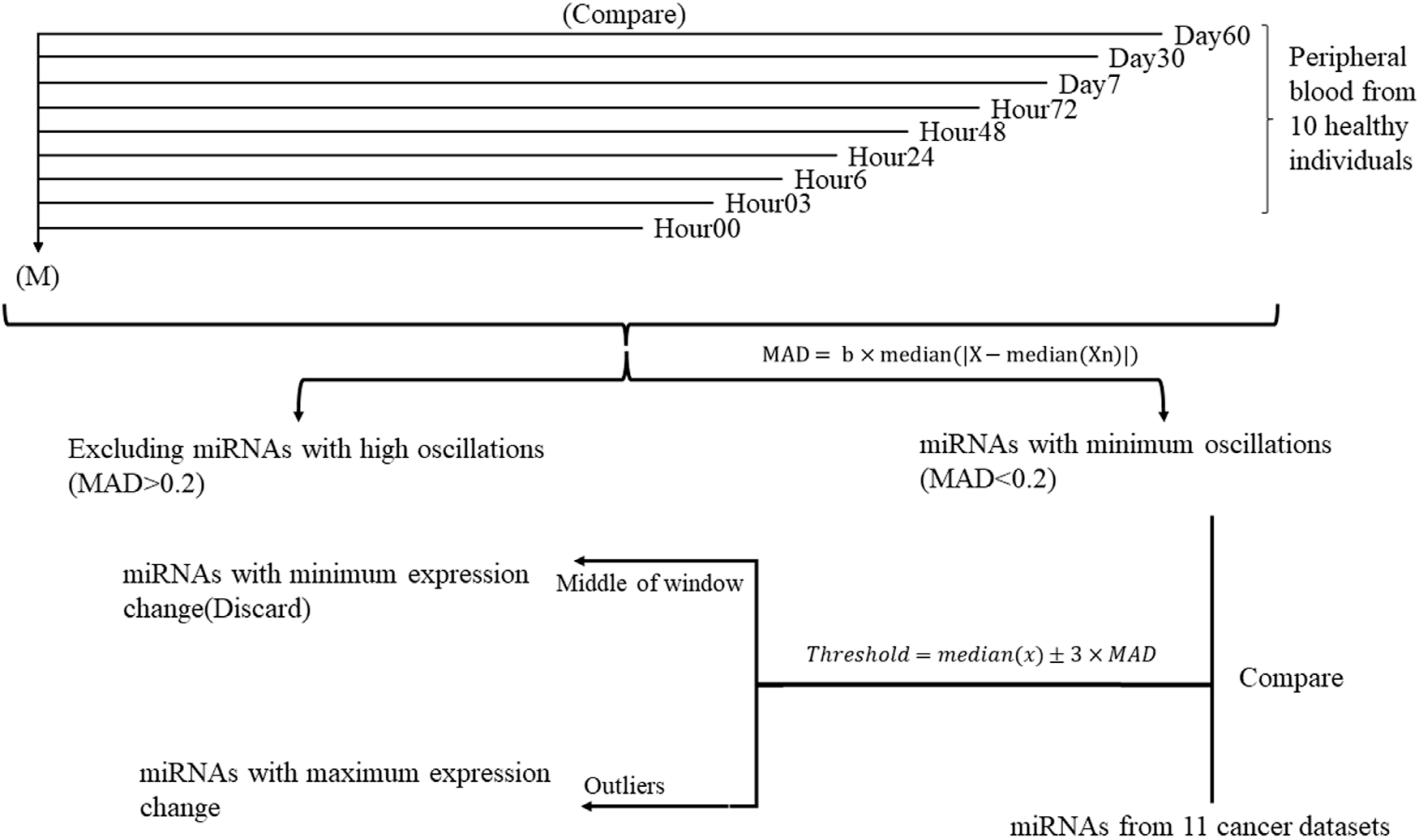
Study design flowchart performed on ten healthy individuals throughout nine time points. Each time point is compared to the mean of expression of 90 samples during nine timepoints (M) separately. miRNAs with similar expression in all time points were selected for further analysis.

This study was conducted in two steps (Figure 1). At first, to determine the miRNAs with minimum oscillation in different time points, each time point’s Deseq2-normalized count is compared to the mean of Deseq2-normalized count in all nine time points separately. miRNAs that showed aberrant expression across time points were therefore removed from the analysis. Median absolute deviation (MAD) was applied to measure miRNA variability fold changes during nine time points and define the cutoff for expression change (Rousseeuw and Croux, 1993; Leys et al., 2013). The value of MAD for each miRNA was calculated as follows, where *b* is constant (*b* = 1.4826) (Leys et al., 2013) and X_n_ is the expression value Log2(FC) for a single miRNA of each time point of healthy samples compared to the overall mean expression:

$$MAD=b\times median\left(\left|X-median\left(X\right)\right|\right)$$ (1)

To specify whether the miRNA was diverted throughout the timepoints, thresholds were defined as where x stands for each miRNA in all time points:

$$Median\left(x\right)=\pm \;3\times MAD$$ (2)

In the second step, miRNAs with MAD <0.2 and fold changes out of the threshold range (outlier) compared to cancer datasets and their specific control samples were selected for further analysis. COSINOR analysis was used to confirm the absence of oscillation in selected miRNAs.

### Data processing and differential expression analysis

Small RNA-seq data analysis was performed on the GALAXY platform (Afgan et al., 2016). The quality of the small RNA libraries was assessed using FASTQC software. Sequence quality was checked per base sequence quality, and all reads with a mean quality score under 30 were excluded. Trim Galore was used to discard adapter sequences and sequences less than 16 bases to ignore false positive mapping. Then sequences were aligned to the human reference genome (Hg38). The annotation file was used from miRbase v22.1 (Ambros et al., 2003), which contains all known human miRNAs. Alignment and quantification were performed using Bowtie 1.2.0 and FeatureCount 1.6.4 (Bates et al., 2014; Mascher et al., 2017). Differential expression analysis of miRNAs was performed using DESeq2 version 2.11.40.6 with the Benjamini–Hochberg procedure, which controls the false discovery rate (FDR) (Love et al., 2016). miRNAs with DESeq2-normalized mean read counts under 20 were discarded to avoid false-positive results.

### Cluster analysis of gene expression

TimeClust software (Magni et al., 2008) was used to cluster the miRNA’s expression foldchanges during nine time points according to their temporal expression profile. The random walk models for Bayesian clustering were used as the clustering algorithm.

Principal component analysis (PCA) was performed using GraphPad Prism version 9.0.0 (GraphPad Software, San Diego, California United States, www.graphpad.com) to specify clustering in healthy *versus* cancer samples.

### ROC curve analysis

The Deseq2-normalized read counts of all overlapping differentially expressed miRNAs(DEMs) were used to assess the sensitivity and specificity of each DEM to evaluate its detection capability in cancer datasets *versus* the control group using receiver-operating characteristic (ROC) curves and the area under curve (AUC).

The sensitivity and specificity of all miRNAs as a prognostic signature for prediction were evaluated by AUC of the receiver operation characteristic with R program software using the Combiroc package (Bombaci and Rossi, 2019).

### Functional enrichment analysis

MIENTURNET (Licursi et al., 2019) was used to find the interaction network of miRNAs with their target genes using Targetscan (McGeary et al., 2019) and miRTarbase (Huang et al., 2022) database and exploring the Kyoto encyclopedia of genes and genomes (KEGG) database (Kanehisa and Goto, 2000). Also, we used ShinyGo as a powerful functional enrichment analyzer tool to specify the KEGG pathway and Gene ontology of targeted genes (Ge et al., 2020).

### miRNA-protein interaction network

The MiRNET platform was used to explore the miRNA target genes and the miRNA-gene interaction network (Chang et al., 2020). Moreover, we explored other miRNA target prediction analyzing tools, including miRDIP(Tokar et al., 2018), miRDB (Chen and Wang, 2020), TargetScan, and miRTarBase by a Venn diagram to find shared miRNAs. Each miRNA and target gene with interaction number <3 (Degree filter) were discarded to reduce the false positive targets.

### Validation

The expression of candidate miRNAs was explored in three different studies. Array express and TCGA datasets was assessed and samples obtained from whole blood of patients with different types of cancer with no treatment was selected. Three different study were explored and analyzed. miRNA expression level from whole blood of patients detected by lung cancer and nasopharyngeal cancer including 1052 healthy individuals and 1438 cancer patients were assessed (Patnaik et al., 2012; Wen, 2018; Fehlmann et al., 2020). The mean expression levels of the seven candidate miRNAs in the blood of cancer patients was compared to the mean expression levels in blood of healthy individuals. The expression intensity of candidate miRNAs in the blood of cancer patients demonstrated a significant difference with expression levels in healthy blood (Figure 8) (Supplementary File S1). All datasets were analyzed by one-way ANOVA followed by Dunnett’s multiple comparisons tests using GraphPad Prism version 9.0.0 for Windows, GraphPad Software, San Diego, California United States, www.graphpad.com.

## Results

### miRNAs have different oscillation patterns

**FIGURE 2:**
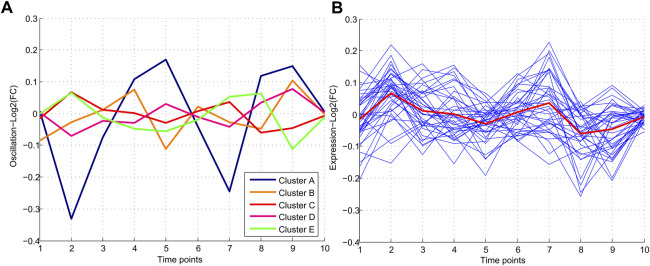
Patterns of miRNA expression during nine different time points. **(A)** Five clusters were detected in clustering analysis. Pattern C which includes 37 miRNAs, demonstrates the minimum and Pattern A demonstrated the maximum oscillation among the other patterns. **(B)** Pattern C consisting of 37 miRNAs with a similar oscillation pattern which demonstrated the lowest oscillations.

The common miRNAs through nine time points were analyzed to find the miRNAs with minimum oscillation patterns. One hundred ninety-five miRNAs were found that were expressed in all time points (Supplementary File S1). Five different oscillation patterns were detected (Figure 2). Cluster E comprised most miRNAs (61), and Cluster A the least (17). Cluster C (red line) comprised 37 miRNAs with the lowest MAD score (MAD <0.2) among other clusters and showed the most consistent behavior. Hence cluster C represented a group of miRNAs with minimum fluctuation in expression levels across all time points (Supplementary File S1).

### miRNAs with minimum oscillation patterns show dysregulation in cancers

**FIGURE 3:**
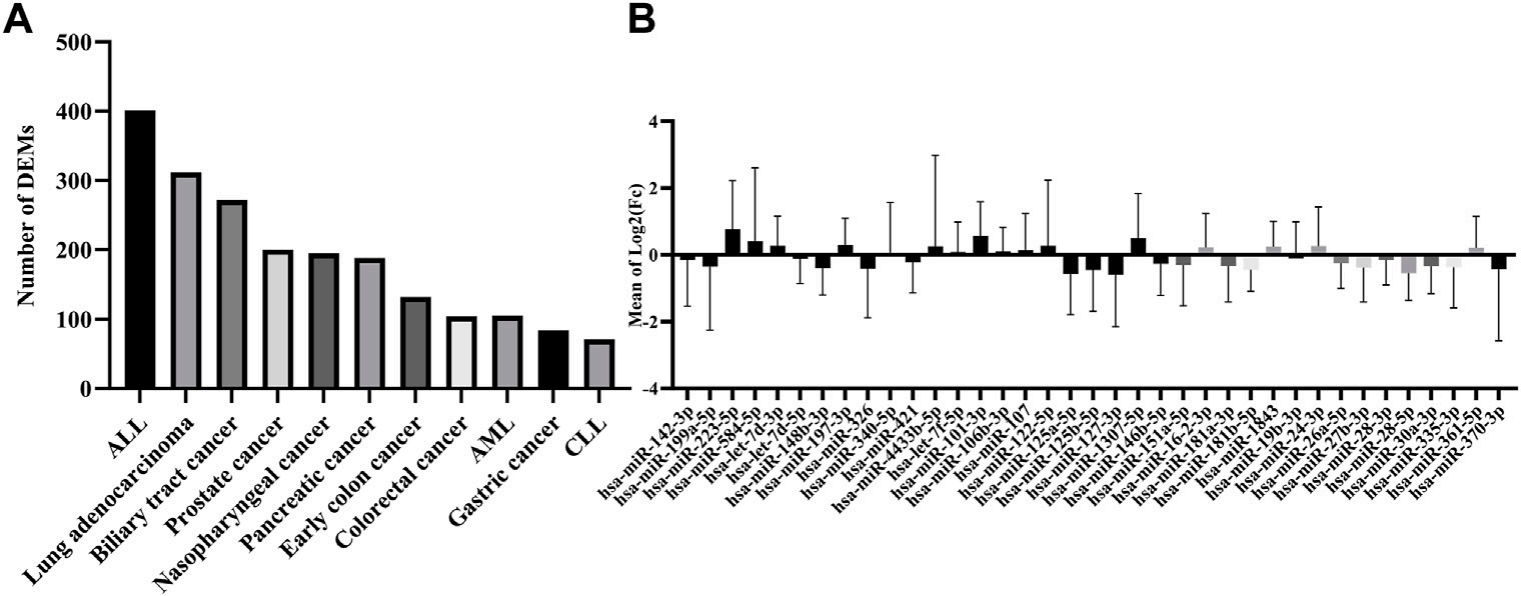
Expression and number of miRNAs in all cancer datasets. **(A)** The absolute number of miRNAs were differentially expressed in each cancer dataset. **(B)** The range of expression levels of each miRNA in all cancer datasets.

To evaluate the potential dysregulation regarding miRNAs in cluster C (Figure 2A), we explored the total number of differentially expressed miRNAs (DEM) in the blood of 11 cancers in comparison to their study-specific controls (Figure 3A). The range of DEMs varied from 401 in ALL (Acute lymphocyte leukemia) to 74 in CLL (Chronic lymphocyte leukemia). The fold changes of 37 candidate miRNAs (cluster C) in all cancers indicated that 17 miRNAs with positive and 20 miRNAs with a negative average of fold changes throughout all 11 cancer datasets. The miR-223-5p demonstrated the highest (1.5), and miR-19b-3p showed the lowest (0.01) average of fold changes compared to study-specific controls (Figure 3B).

Finally, the presence (miRNA with Adj *p*-value <0.05) and dysregulation level of the 37 candidate miRNAs were checked in all types of cancer by ranking miRNAs according to their distance from the threshold window. The top seven DEMs were selected based on their distance from the threshold (outliers) [2], including miR-142-3p, miR-199a-5p, and miR-223-5p showed the most significant distance to the cutoff border. Seven miRNAs with significantly distinct fold change in comparison to cancer datasets and study-specific controls and also distance from the threshold window in more than 80% of cancers were selected as top dysregulated miRNAs.

**FIGURE 4:**
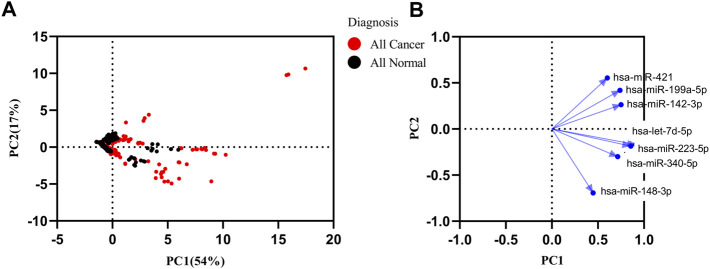
Principal component analysis (PCA) of healthy and all types of cancer. **(A)** PCA matrix was used to discover cancer and control group. 71% variance was discovered in PC1 and PC2. **(B)** Loading plot of seven miRNAs.

In order to find the miRNAs responsible for variance in expression of the cancer datasets from healthy and cancer samples, we performed principal component analysis (PCA) with the first principal component (PC1) and second principal component (PC2) for seven miRNAs. The PC1 showed 54%, and PC2 showed a 17% (Figure 4A) variance between all samples. A loading plot was created utilizing the PC axis to distinguish between the miRNAs responsible for these clusters. On the PC2, let-7d-5p, miR-148b-3p, miR-223-5p, and miR-340-5p all exhibited a negative value. miR-148b-3p and miR-421 were small contributors to the PC1. The PC2 contained the highest contribution levels for miR-148b-3p and miR-421 (Figure 4B). The panel of miRNAs demonstrated the best discrimination power in lung, Biliary tract and nasopharyngeal cancer.

Moreover, we conducted a literature review to compare the reported miRNAs in other cancer studies with the present candidate miRNAs result (Supplementary File S2). The most important miRNAs reported numerously as biomarkers in the blood of cancers were miR-142-3p and miR-223-5p. These miRNAs and other reported miRNAs demonstrated significant dysregulation in different cancer types.

### Diagnostic values of DEMs

**FIGURE 5:**
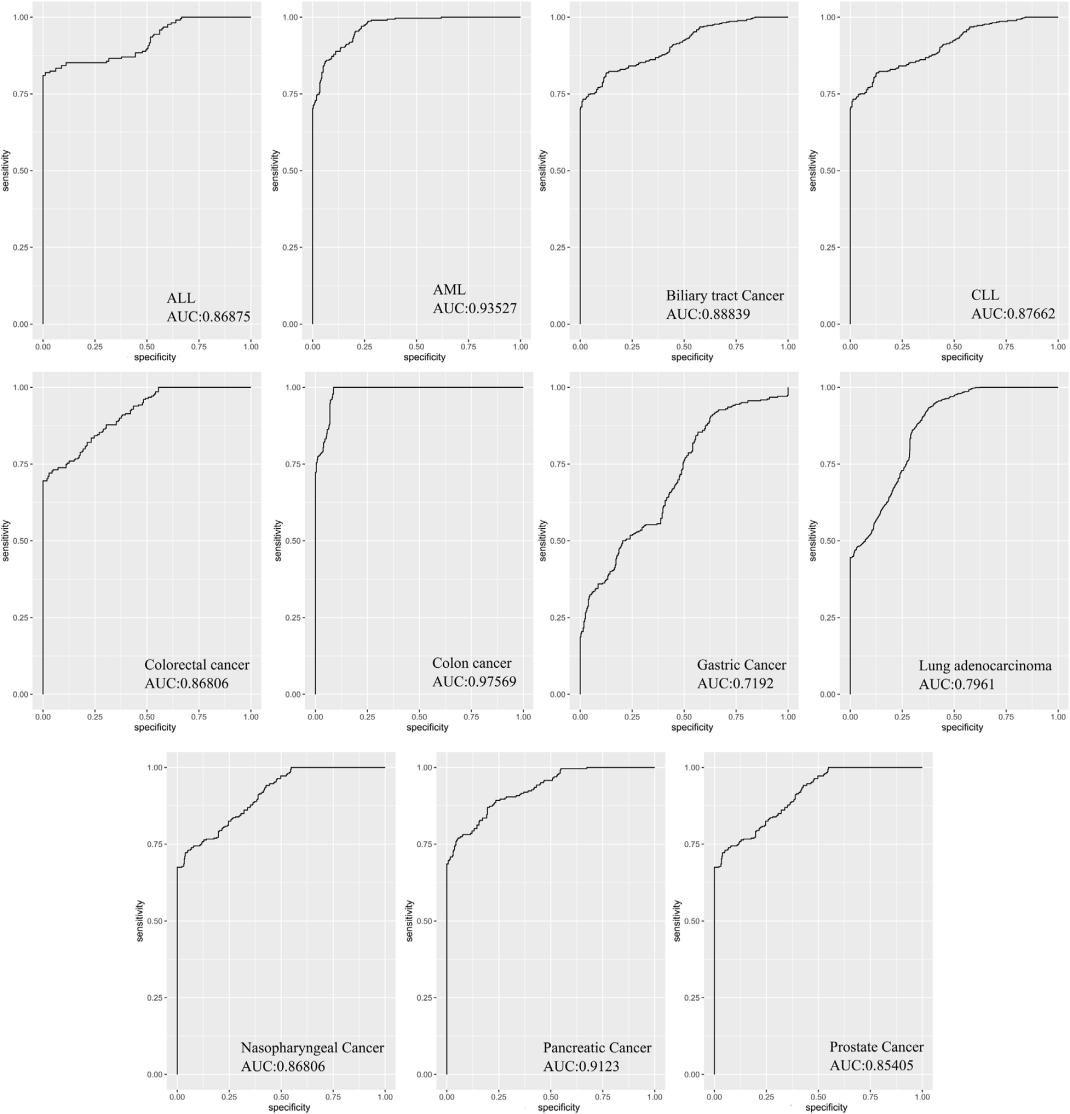
The area under curves analysis for seven DEMs as a signature in eleven cancers. AUC analysis of all seven miRNAs as a signature panel checked with sensitivity and specificity >80% and *p*-value <0.05. The best model performance was demonstrated in Colon cancer.

In order to evaluate the diagnostic values of DEMs in discriminating cancer from healthy controls, ROC curve analysis was performed on Deseq2-normalized read counts of seven miRNAs with minimum oscillation. The seven miRNAs displayed promising results in discriminating the two groups with a specificity and sensitivity greater than 80% and a *p*-value <0.05 (Supplementary File S1). The best model performance was observed in AML, pancreatic, and colon cancers (AUC> 90%). Gastric cancer and lung adenocarcinoma showed lower performance (AUC>80%) than other cancers. Seven miRNAs, including miR-142-3p, miR-199a-5p, miR-223-5p, let-7d-5p, miR-148b-3p, miR-340-5p, and miR-421 demonstrated the significant signatures as a panel for distinguishing cancer from healthy samples (Figure 5).

### miRNA-protein interaction network and functional analysis

**FIGURE 6:**
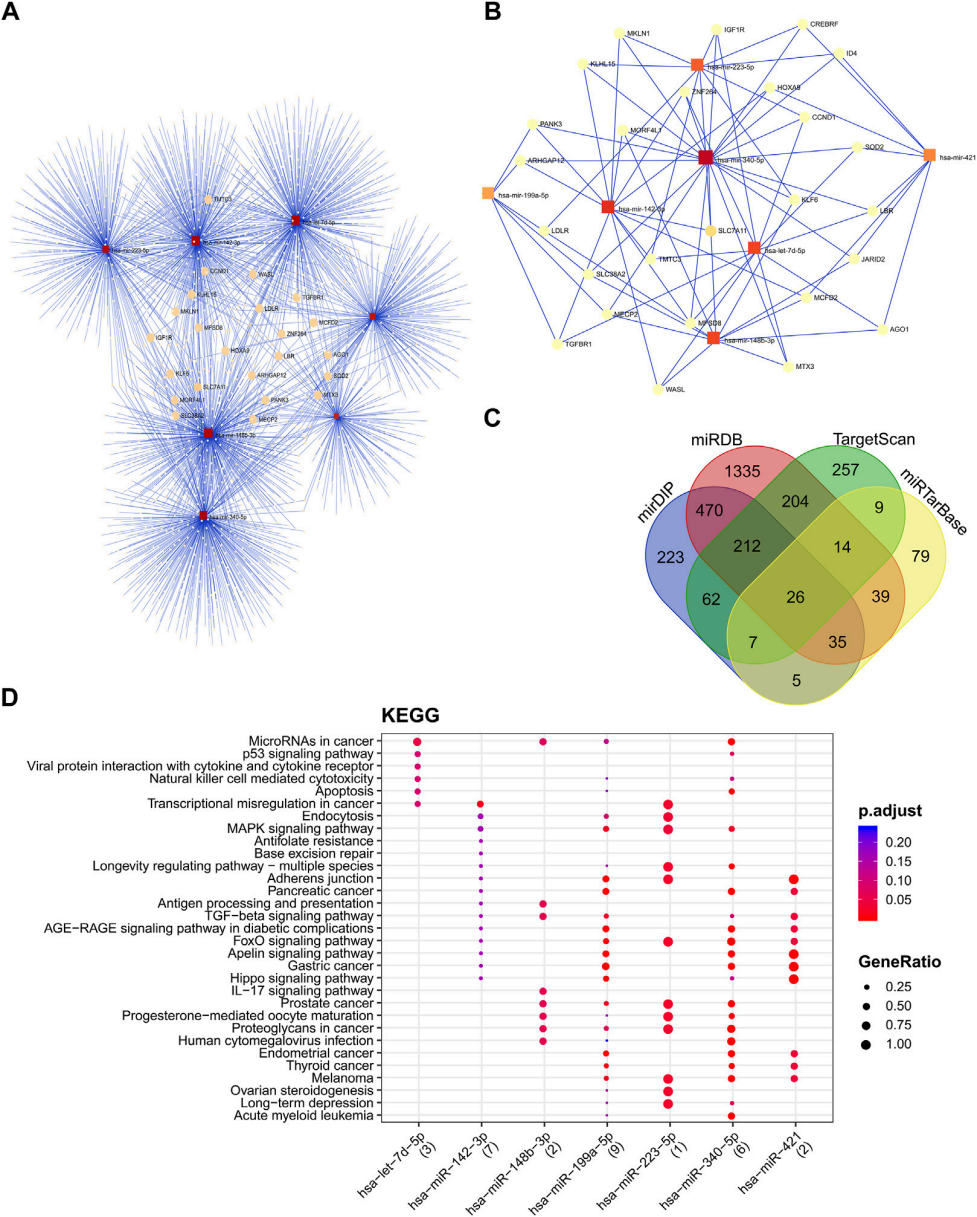
The miRNA-protein interaction network and cluster analysis of target genes and the interactions between selected miRNAs and target genes. **(A)** miRNA-mRNA interaction network of all seven candidate miRNAs. **(B)**The best miRNA candidate association with target genes squares represented miRNAs, and circles represented target genes. **(C)** miRNA target prediction tools were explored and found 26 best-fitted target genes common in all platforms. **(D)** KEGG enrichment analysis of DEMs. KEGG pathway analysis showed candidate miRNAs significantly implicated with the cancer-related disease.

The miRNA interaction network of seven candidate miRNAs had 2212 edges (interactions) and 1971 nodes (target genes) (Figure 6A). Several miRNA target predictions analyzing were used to find the best target genes for each miRNA. A module with 26 nodes and 79 edges was extracted with the most significant interaction scores (degree score ≥3), including seven miRNAs and 26 target genes (Figure 6B). The list of best fitted genes is available in Supplementary File S1 and Supplementary Table S5. The miR-340-5p and miR-142-3p demonstrated the best interaction by having the most connectivity with target genes (Figure 6C). The KEGG pathway enrichment analysis of candidate miRNAs showed that microRNAs in cancer and P53 signaling pathway are the most important pathways (Figure 6D).

**FIGURE 7:**
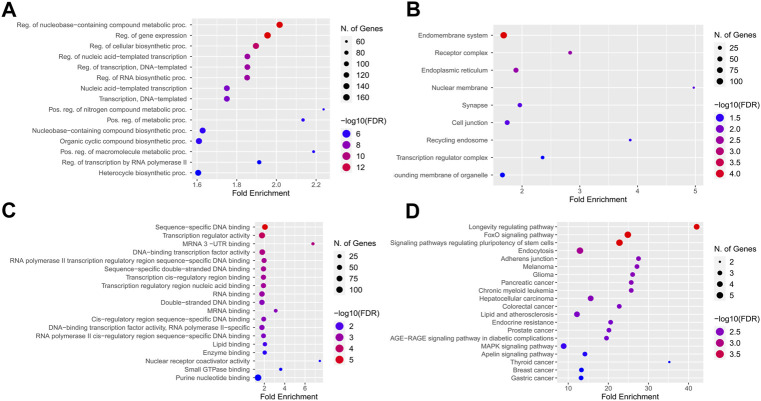
KEGG pathway enrichment and Gene ontology analysis best-fitted target genes. **(A)** Biological process term (BP) association with nucleobase containing compound metabolite process and regulation of gene expression. **(B)** Endomembrane system of cells were the cellular components (CC) most involved regarding targeted genes. **(C)** In molecular function (MF) categories, target genes are associated with sequence-specific DNA binding, and transcription regulatory activity. **(D)** Longevity regulation pathway, FOXO signaling pathway and signaling pathways regulation pluripotency of stem cells had best enrichment ratio and the more significant number of identified targets in KEGG pathway analysis.

The KEGG pathway enrichment and gene ontology analysis on 26 best-fitted target genes were performed using the ShinyGO tool. GO analysis in biological process term (BP) demonstrated that the target genes were associated with nucleobase-containing compound metabolite process and regulation of gene expression (Figure 7A). In addition, the endomembrane system was the cellular components (CC) most involved in related targeted genes (Figure 7B). In molecular function (MF) categories, target genes are associated with sequence-specific DNA binding and transcription regulatory activity. miRNA prediction tools were explored to find the overlap (Figure 7C). On the other hand, we checked the KEGG enrichment results based on their weight scores to cluster results based on the minimum subset of target genes that cover all the genes from enrichment sets. FOXO signaling pathway, signaling pathways regulating pluripotency of stem cells, and longevity regulation pathway were demonstrated with a greater fold enrichment ratio and significant FDR <0.05 (Figure 7D).

### Validation

**FIGURE 8:**
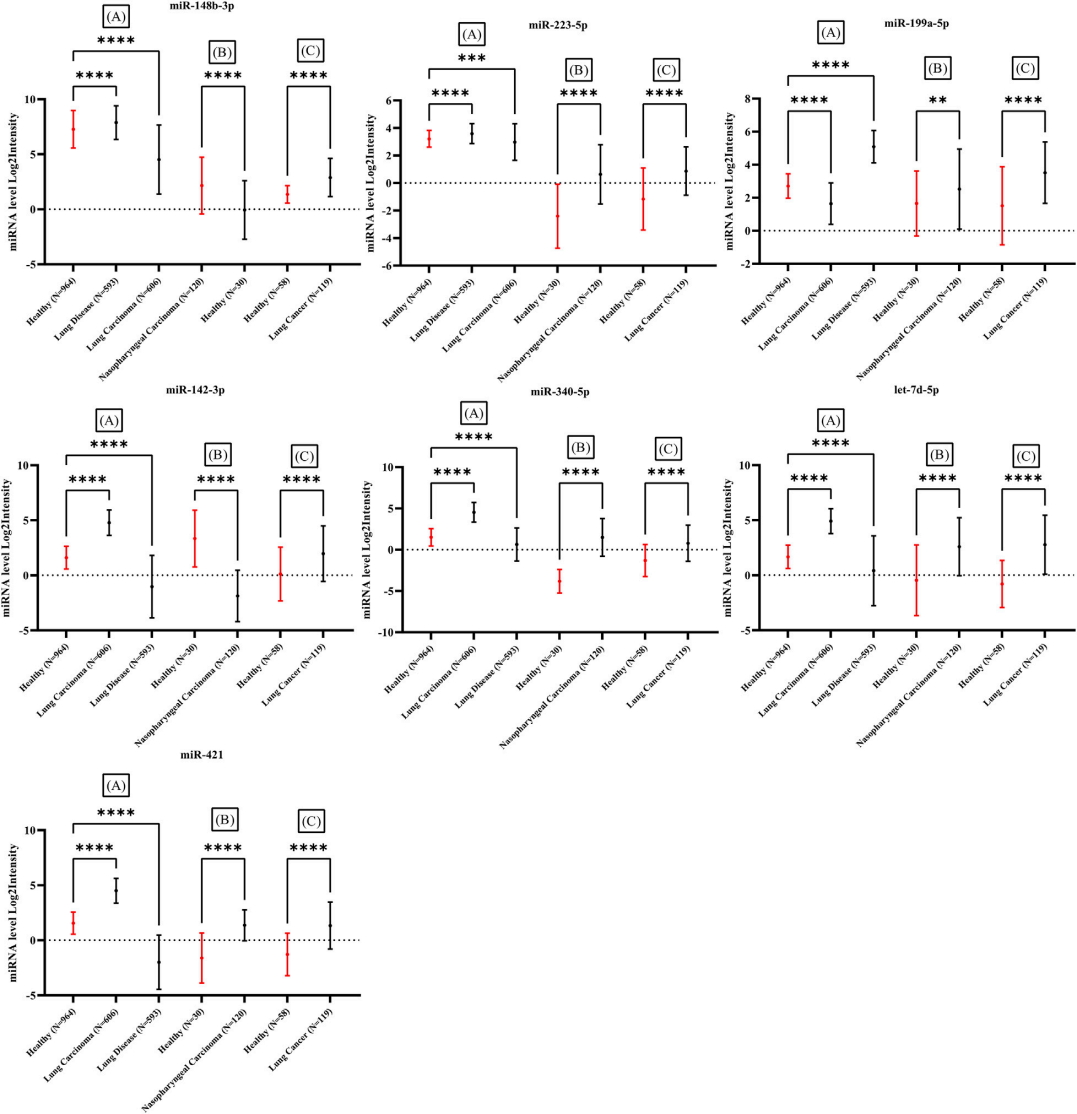
Expression difference in seven miRNAs in comparison between healthy individuals and several types of cancer in three studies. All samples obtained from whole blood of cancer patients. Significant difference demonstrated with *p*-value <0.05 in all three studies. Three studies including (A): (Fehlmann et al., 2020), (B): (Wen, 2018) and (C): (Patnaik et al., 2012).

The expression of candidate miRNAs was explored in total seven studies Including three studies from different cancer types obtained from whole blood. The expression intensity of candidate miRNAs in the whole blood (Figure 8) of cancer patients demonstrated a significant difference in compare the healthy controls. Statistical analysis provided as supplementary file (Supplementary File S1 and Supplementary Table S6). All clinical data including sex and age of control and patient samples were provided in supplementary file (Supplementary File S1 and Supplementary Table S7).

## Discussion

The molecular mechanisms that control a range of genes across time are part of the human biological clock, which influences many molecular processes (Takahashi et al., 2008). The regulation of the daily timing of the transcriptome, including miRNA, is organized by the circadian cycle (Du et al., 2014; Zhou et al., 2021). As a result, one of the key variables influencing the results of gene expression investigations is the sampling time. In the present study, we have tried to find miRNAs in the peripheral blood with minimum fluctuation at different time points. Then, the candidate miRNAs expression was assessed in eleven cancers.

The blood miRNAs in healthy controls showed different oscillation patterns. We have found a cluster of 37 miRNAs with the most stable behavior across all time points. It can be assumed that a change in the expression of these miRNAs may indicate an abnormal or pathological event (Farrell et al., 2015; Bertoli et al., 2016). Therefore, we explored the expression level of these stable miRNAs in the peripheral blood of different types of cancer. We have found that seven miRNAs, including miR-142-3p, miR-199a-5p, miR-223-5p, let-7d-5p, miR-148b-3p, miR-340-5p, and miR-421 were significantly deregulated in the blood of cancer patients. The sensitivity and specificity of these miRNAs were higher than 80%. Several studies have been reported the deregulation of miR-142-3p (Lv et al., 2012; Hu et al., 2013; Wang et al., 2018; Gao et al., 2019; Liu et al., 2021), miR-199a-5p (Zhou et al., 2015; Zhang et al., 2021), miR-223-5p (Gilicze et al., 2014; Zhu et al., 2019; Deng et al., 2021), let-7d-5p (De Santis and Götte, 2021; Li et al., 2021), miR-148b-3p (Mollazadeh et al., 2019; Yuan et al., 2019; Shan et al., 2021), miR-340-5p (Guo et al., 2021; Huang et al., 2021; Tan et al., 2021), and miR-421 (Chen et al., 2019; Mo et al., 2020) in the blood of different cancer types. The KEGG pathway analysis showed that the microRNAs in cancer and P53 signaling pathway were the most enriched pathways. These pathways are important routes of cancer development and progression.

The critical question is how changes in the expression of miRNAs in the blood may be associated with cancer in other tissues. A standard answer to this question is that the leakage of miRNAs from cancerous tissues into the blood changes the level of these molecules in the blood of cancer patients (Fichtlscherer et al., 2011; Keller et al., 2011; Creemers et al., 2012). However, blood cells are the primary producer of circulating miRNA (Pritchard et al., 2012; Sohel, 2016). Moreover, comparisons of blood miRNAs with cancerous tissues have shown that miRNAs in blood are not just a byproduct of cancerous tissue but are a part of the body’s defense against cancer (Coombs et al., 2017).

In a contradictory hypothesis (a version of clonal hematopoiesis) (Jamieson, 2017), it can be assumed that deregulation of circulating miRNAs due to somatic mutation in hematopoietic stem cells may lead to an increased risk of cancer in other tissues. It has been shown that miRNAs can be transferred to cancer cells and regulate cellular processes and signaling pathways (Turchinovich et al., 2011; Ramachandran and Palanisamy, 2012; Turchinovich and Burwinkel, 2012; Turchinovich et al., 2013; Chevillet et al., 2014; Turchinovich et al., 2015; Anfossi et al., 2018; Sun et al., 2018; Reshke et al., 2020). We have found seven miRNAs that are highly stable under normal conditions, which may dysregulate in mutated hematopoietic cells with age (Coombs et al., 2017; Jamieson, 2017; Fabre et al., 2022; Jeon et al., 2022). In addition, this panel of seven miRNA may have use in surgical treatment response. There are numerous studies reporting the use of circulating miRNAs in surgical treatment response. Recent studies have shown that the expression of some circulating miRNAs in patients diagnosed with cancer was back to the level of expression in healthy individuals after surgical treatment (Heneghan et al., 2010; van Schooneveld et al., 2012; Chen et al., 2016). Although the present study results are promising for cancer detection, there is a limitation due to using samples in different stages. Therefore, it is not clear whether these miRNAs could detect cancer in its early stages. Furthermore, due to the lack of time series datasets in healthy individual’s females, the results may include sex bias expression. Also, Deeper verification is required for our findings due to poor overlap among the other reports.

In summary, we have found a pan-cancer signature of seven miRNAs that may have the potential for cancer detection. Also, this approach could be used as a survey for identifying biomarkers for other pathological conditions. However, further investigation is needed to validate our results and examine the miRNA’s pan-cancer role.

## Data Availability

The original contributions presented in the study are included in the article/Supplementary Material, further inquiries can be directed to the corresponding author.
