# Supplementary material for: A panel of blood-derived miRNAs with a stable expression pattern as a potential pan-cancer detection signature
Source: Front Mol Biosci. 2022 Dec 15;9:1030749. doi: 10.3389/fmolb.2022.1030749 (PMC9798419; doi:10.3389/fmolb.2022.1030749)
Supplement: Supplementary file 1 [file DataSheet1.docx]

**Supplementary 1**

**Table S1:** Table of clinical information of 10 healthy individuals. All the participants were non smokers and free of all medication during the study course(Mussack et al., 2021).

| **Study subject** | **1** | **2** | **3** | **4** | **5** | **6** | **7** | **8** | **9** | **10** |
| --- | --- | --- | --- | --- | --- | --- | --- | --- | --- | --- |
| **Age** | 34 | 36 | 25 | 29 | 26 | 28 | 26 | 28 | 26 | 25 |
| **Weight (Kg)** | 83 | 83 | 85 | 78 | 92 | 88 | 78 | 90 | 76 | 78 |
| **Height(M)** | 1.81 | 1.83 | 1.92 | 1.80 | 1.80 | 1.85 | 1.90 | 1.86 | 1.78 | 1.82 |
| **Physical Activity (Hours/Week)** | 3 | 3.5 | 3.5 | 4.5 | 2.5 | 2.5 | 2.5 | 6 | 2.5 | 3 |

**Table S2**: MAD score of 195 common miRNAs in nine healthy datasets.

| **miRNA** | **MAD** | **miRNA** | **MAD** | **miRNA** | **MAD** | **miRNA** | **MAD** | **miRNA** | **MAD** | **miRNA** | **MAD** |
| --- | --- | --- | --- | --- | --- | --- | --- | --- | --- | --- | --- |
| hsa-miR-744-5p | 0.018914 | hsa-let-7f-1-3p | 0.066879 | hsa-miR-320b | 0.097815 | hsa-miR-1908-5p | 0.120184 | hsa-miR-125a-5p | 0.147419 | hsa-miR-101-3p | 0.323184 |
| hsa-miR-941 | 0.0227 | hsa-miR-25-3p | 0.067489 | hsa-miR-222-3p | 0.098248 | hsa-miR-1843 | 0.121029 | hsa-miR-26a-5p | 0.147964 | hsa-miR-96-5p | 0.328066 |
| hsa-miR-148b-3p | 0.023327 | hsa-miR-324-5p | 0.068093 | hsa-miR-323b-3p | 0.098918 | hsa-miR-25-5p | 0.121773 | hsa-miR-423-5p | 0.14901 | hsa-miR-340-5p | 0.359542 |
| hsa-miR-186-5p | 0.026294 | hsa-miR-18a-3p | 0.068448 | hsa-miR-28-3p | 0.098956 | hsa-miR-769-5p | 0.121915 | hsa-miR-484 | 0.149915 | hsa-miR-142-3p | 0.541931 |
| hsa-miR-6802-3p | 0.027149 | hsa-miR-6505-3p | 0.068553 | hsa-miR-16-2-3p | 0.099695 | hsa-miR-4473 | 0.122219 | hsa-miR-618 | 0.150558 | hsa-miR-122-5p | 0.694104 |
| hsa-miR-335-3p | 0.027832 | hsa-miR-140-3p | 0.068769 | hsa-miR-576-5p | 0.100186 | hsa-miR-320c | 0.122714 | hsa-miR-200c-3p | 0.152339 |  |  |
| hsa-miR-30e-3p | 0.028239 | hsa-miR-193a-5p | 0.07096 | hsa-miR-24-3p | 0.100361 | hsa-miR-1229-3p | 0.125476 | hsa-miR-3913-5p | 0.152371 |  |  |
| hsa-miR-22-3p | 0.029504 | hsa-miR-345-5p | 0.07133 | hsa-let-7d-3p | 0.10053 | hsa-miR-1284 | 0.126499 | hsa-miR-550a-3-5p | 0.153422 |  |  |
| hsa-miR-29c-5p | 0.031012 | hsa-miR-6501-5p | 0.073279 | hsa-miR-6881-3p | 0.101588 | hsa-miR-6726-3p | 0.127962 | hsa-miR-1976 | 0.154138 |  |  |
| hsa-let-7a-5p | 0.034788 | hsa-miR-942-5p | 0.073855 | hsa-miR-1306-5p | 0.101715 | hsa-miR-3173-5p | 0.128101 | hsa-miR-30e-5p | 0.154463 |  |  |
| hsa-let-7c-5p | 0.037588 | hsa-miR-3200-5p | 0.074374 | hsa-miR-4732-3p | 0.104839 | hsa-miR-125b-5p | 0.128442 | hsa-miR-485-3p | 0.162155 |  |  |
| hsa-miR-106b-3p | 0.038177 | hsa-miR-197-3p | 0.076736 | hsa-miR-1249-3p | 0.106634 | hsa-miR-363-3p | 0.128906 | hsa-miR-7-5p | 0.168417 |  |  |
| hsa-miR-155-5p | 0.040768 | hsa-miR-330-3p | 0.077558 | hsa-miR-1307-5p | 0.108256 | hsa-miR-451a | 0.129094 | hsa-miR-378a-3p | 0.173752 |  |  |
| hsa-miR-5189-3p | 0.044552 | hsa-miR-629-5p | 0.078716 | hsa-miR-4326 | 0.108518 | hsa-miR-500a-3p | 0.130066 | hsa-miR-1260b | 0.175039 |  |  |
| hsa-miR-150-5p | 0.050408 | hsa-miR-331-3p | 0.079032 | hsa-miR-191-5p | 0.108692 | hsa-miR-330-5p | 0.130472 | hsa-miR-4659b-3p | 0.175573 |  |  |
| hsa-miR-425-5p | 0.05068 | hsa-miR-550a-3p | 0.079258 | hsa-miR-326 | 0.109694 | hsa-miR-3200-3p | 0.13077 | hsa-miR-185-5p | 0.180937 |  |  |
| hsa-let-7b-3p | 0.050798 | hsa-miR-23b-3p | 0.079445 | hsa-miR-181a-3p | 0.110263 | hsa-let-7i-5p | 0.131129 | hsa-miR-486-5p | 0.183615 |  |  |
| hsa-miR-92b-3p | 0.051321 | hsa-miR-4659a-3p | 0.0799 | hsa-miR-532-5p | 0.11037 | hsa-miR-30c-5p | 0.131183 | hsa-miR-4742-3p | 0.183671 |  |  |
| hsa-miR-148a-5p | 0.053944 | hsa-miR-424-3p | 0.080839 | hsa-miR-1304-5p | 0.111881 | hsa-miR-148b-5p | 0.131689 | hsa-let-7a-3p | 0.188553 |  |  |
| hsa-miR-654-3p | 0.055331 | hsa-miR-99b-5p | 0.080999 | hsa-miR-6803-3p | 0.1124 | hsa-miR-6511b-3p | 0.132583 | hsa-miR-340-3p | 0.191168 |  |  |
| hsa-miR-339-3p | 0.055585 | hsa-miR-1292-5p | 0.0811 | hsa-miR-324-3p | 0.112404 | hsa-let-7f-5p | 0.132657 | hsa-miR-27b-3p | 0.194526 |  |  |
| hsa-miR-221-3p | 0.055768 | hsa-miR-199a-5p | 0.082682 | hsa-miR-1307-3p | 0.112433 | hsa-miR-10b-5p | 0.134118 | hsa-miR-183-5p | 0.194553 |  |  |
| hsa-miR-182-5p | 0.056104 | hsa-miR-548o-3p | 0.083234 | hsa-miR-454-5p | 0.112555 | hsa-miR-181a-5p | 0.134618 | hsa-miR-98-5p | 0.196265 |  |  |
| hsa-miR-766-3p | 0.057343 | hsa-miR-425-3p | 0.083861 | hsa-miR-107 | 0.114771 | hsa-miR-142-5p | 0.134859 | hsa-miR-139-5p | 0.208078 |  |  |
| hsa-miR-1180-3p | 0.059328 | hsa-miR-181a-2-3p | 0.084227 | hsa-miR-1226-3p | 0.114884 | hsa-miR-6741-3p | 0.134925 | hsa-miR-146b-5p | 0.210768 |  |  |
| hsa-miR-625-3p | 0.059363 | hsa-miR-574-3p | 0.085545 | hsa-miR-185-3p | 0.11495 | hsa-miR-937-3p | 0.135555 | hsa-miR-1468-5p | 0.21333 |  |  |
| hsa-miR-6734-3p | 0.059782 | hsa-miR-1301-3p | 0.086118 | hsa-miR-320a-3p | 0.115403 | hsa-miR-664a-3p | 0.135815 | hsa-miR-151a-5p | 0.21819 |  |  |
| hsa-miR-342-3p | 0.060205 | hsa-miR-6513-3p | 0.086801 | hsa-miR-4521 | 0.115651 | hsa-miR-339-5p | 0.136489 | hsa-miR-532-3p | 0.218307 |  |  |
| hsa-miR-28-5p | 0.060667 | hsa-miR-363-5p | 0.086837 | hsa-miR-4433b-5p | 0.115956 | hsa-miR-1294 | 0.136672 | hsa-miR-4755-5p | 0.220216 |  |  |
| hsa-miR-550a-5p | 0.060943 | hsa-miR-505-5p | 0.087276 | hsa-miR-192-5p | 0.116405 | hsa-miR-505-3p | 0.136759 | hsa-let-7b-5p | 0.235924 |  |  |
| hsa-miR-30d-5p | 0.061496 | hsa-miR-30a-5p | 0.089308 | hsa-miR-6511a-3p | 0.116565 | hsa-miR-502-3p | 0.137609 | hsa-miR-378i | 0.237377 |  |  |
| hsa-miR-152-3p | 0.06248 | hsa-miR-181b-5p | 0.08946 | hsa-miR-15b-3p | 0.116806 | hsa-miR-194-5p | 0.1396 | hsa-miR-148a-3p | 0.238019 |  |  |
| hsa-let-7d-5p | 0.062973 | hsa-miR-30a-3p | 0.093093 | hsa-miR-22-5p | 0.117172 | hsa-miR-18b-3p | 0.1401 | hsa-miR-342-5p | 0.250224 |  |  |
| hsa-miR-26b-3p | 0.063933 | hsa-miR-501-3p | 0.093349 | hsa-miR-92a-3p | 0.119479 | hsa-miR-11401 | 0.14061 | hsa-miR-19b-3p | 0.252063 |  |  |
| hsa-miR-130b-5p | 0.064195 | hsa-miR-584-5p | 0.093381 | hsa-miR-652-3p | 0.119767 | hsa-miR-27a-3p | 0.141238 | hsa-miR-3909 | 0.269844 |  |  |
| hsa-miR-378a-5p | 0.065962 | hsa-miR-625-5p | 0.093797 | hsa-miR-29a-3p | 0.120085 | hsa-miR-1343-3p | 0.144334 | hsa-miR-210-3p | 0.273247 |  |  |
| hsa-miR-361-5p | 0.066111 | hsa-miR-421 | 0.095586 | hsa-miR-4685-3p | 0.12016 | hsa-miR-127-3p | 0.145015 | hsa-miR-370-3p | 0.281683 |  |  |
| hsa-miR-328-3p | 0.066161 | hsa-miR-7976 | 0.096891 | hsa-miR-4732-5p | 0.120182 | hsa-miR-6855-3p | 0.146302 | hsa-miR-223-5p | 0.293564 |  |  |

**Table S3**: Median expression of miRNAs in the blood of healthy samples. The threshold (up and down) and the fold change expression of all cancer datasets were presented.

| **ID** | **Thresh Up** | **Thresh Down** | **AML** | **Biliary** | **CLL** | **Colorectal** | **Colom** | **gas** | **lung** | **Naso** | **Panc** | **prostate** | **ALL** |
| --- | --- | --- | --- | --- | --- | --- | --- | --- | --- | --- | --- | --- | --- |
| hsa-miR-142-3p | -0.056 | -0.622 | -1.491 | -0.840 | 0.519 | -0.773 | -0.782 | 2.796 | -1.678 | 0.296 | -0.687 | -0.792 | 1.697 |
| hsa-miR-199a-5p | 0.002 | -0.111 | 2.240 | -2.165 | -3.271 | -0.899 | -0.333 | 1.069 | -2.151 | 2.906 | -1.070 | -0.679 | 0.432 |
| hsa-miR-223-5p | -0.024 | -0.326 | -1.924 | 2.470 | -0.004 | -0.386 | -0.042 | 1.550 | 0.968 | 1.684 | 1.496 | -0.333 | 3.024 |
| hsa-let-7d-5p | 0.082 | -0.263 | 0.623 | -0.435 | -0.967 | -0.676 | 0.559 | 1.473 | -0.876 | 0.162 | -0.222 | -0.271 | -0.623 |
| hsa-miR-148b-3p | 0.008 | -0.097 | 0.005 | -0.834 | -1.409 | -0.666 | -0.233 | -0.841 | -1.400 | 1.248 | -0.675 | -0.135 | 0.592 |
| hsa-miR-340-5p | 0.057 | -0.143 | -2.075 | 1.286 | 0.533 | 0.169 | -1.467 | -0.769 | -1.948 | 1.525 | 0.915 | -0.080 | 2.611 |
| hsa-miR-421 | -0.018 | -0.354 | -0.452 | 1.276 | -1.172 | 0.368 | -0.885 | -1.709 | -0.680 | 0.441 | 0.990 | -0.083 | -0.533 |
| hsa-let-7d-3p | 0.028 | -0.087 | -0.369 | 0.796 | -0.395 | -0.542 | 0.679 | -0.741 | 1.933 | 1.466 | 0.625 | -0.067 | -0.386 |
| hsa-miR-326 | 0.013 | -0.176 | -0.311 | -1.972 | -1.936 | -0.992 | 1.067 | 0.417 | -2.577 | 2.270 | -1.199 | 0.246 | 0.413 |
| hsa-miR-4433b-5p | 0.035 | -0.270 | NA | -2.085 | -3.557 | 0.233 | 0.659 | 1.250 | -0.958 | 4.544 | -1.663 | -0.734 | 4.857 |
| hsa-let-7f-5p | 0.004 | -0.620 | 0.187 | 1.248 | -0.424 | 0.073 | 0.001 | 1.430 | -1.740 | -0.666 | 0.868 | -0.331 | 0.254 |
| hsa-miR-101-3p | 0.001 | -0.205 | 1.286 | 1.223 | 1.639 | -0.388 | -0.286 | 0.322 | 0.133 | -1.095 | 0.846 | 0.056 | 2.433 |
| hsa-miR-106b-3p | 0.004 | -0.399 | 0.545 | 1.441 | 0.054 | 0.060 | 0.659 | -0.064 | -0.415 | -1.021 | 0.857 | -0.285 | -0.752 |
| hsa-miR-107 | -0.009 | -0.161 | -0.179 | 1.119 | -0.108 | -0.774 | 0.268 | 2.375 | -1.180 | -1.424 | 0.940 | 0.122 | 0.435 |
| hsa-miR-122-5p | 0.010 | -1.616 | NA | -1.695 | 0.097 | 0.957 | -1.145 | 0.055 | 4.974 | -0.719 | -1.768 | 0.587 | 1.328 |
| hsa-miR-125a-5p | 0.043 | -0.379 | 0.973 | -2.318 | -2.717 | -0.004 | -0.018 | -1.254 | 0.908 | 0.542 | -1.162 | -0.454 | -0.754 |
| hsa-miR-125b-5p | -0.034 | -0.212 | 1.113 | -2.136 | -0.133 | NA | 0.196 | -1.069 | 1.596 | -1.741 | -0.668 | -0.034 | -1.667 |
| hsa-miR-127-3p | 0.007 | -0.398 | -0.156 | -2.487 | -2.409 | -0.293 | 1.000 | -0.797 | -1.867 | 2.652 | -1.934 | 0.103 | -0.361 |
| hsa-miR-1307-5p | -0.221 | -2.303 | -1.417 | 3.227 | 0.452 | -0.834 | 1.043 | 0.951 | -0.693 | -0.275 | 1.042 | 0.182 | 1.881 |
| hsa-miR-146b-5p | -0.031 | -0.375 | -1.446 | -0.330 | 0.005 | -0.200 | -0.660 | 1.419 | -1.286 | -0.652 | -0.743 | -0.568 | 1.455 |
| hsa-miR-151a-5p | 0.000 | -0.377 | 0.975 | -0.841 | -2.111 | 0.332 | 0.410 | 0.054 | -1.724 | 1.953 | -0.813 | -0.120 | -1.424 |
| hsa-miR-16-2-3p | -0.035 | -0.749 | 1.081 | 0.657 | -0.670 | 0.753 | -0.495 | 1.063 | 1.947 | -0.927 | 0.601 | -0.192 | -1.335 |
| hsa-miR-181a-3p | 0.028 | -0.292 | -1.821 | -0.684 | -1.789 | -0.406 | 0.508 | 0.289 | -1.625 | 1.633 | -0.215 | 0.185 | 0.245 |
| hsa-miR-181b-5p | -0.028 | -0.470 | -1.414 | -0.470 | -1.711 | -0.473 | 0.183 | 0.295 | -0.630 | 0.041 | -0.293 | 0.152 | -0.663 |
| hsa-miR-1843 | 0.045 | -0.390 | 0.736 | 0.488 | -0.811 | -0.397 | 0.559 | 0.937 | -0.771 | 1.519 | 0.433 | -0.474 | 0.522 |
| hsa-miR-19b-3p | -0.013 | -0.256 | -1.141 | 1.150 | 0.079 | -0.603 | -1.115 | -0.041 | 0.886 | -1.135 | 0.637 | -0.163 | 1.647 |
| hsa-miR-24-3p | -0.008 | -0.418 | -0.730 | 2.440 | 0.432 | 0.041 | 0.172 | -0.428 | -1.042 | -1.239 | 1.415 | 0.130 | 1.726 |
| hsa-miR-26a-5p | -0.029 | -0.424 | 0.053 | -0.162 | 0.412 | -0.307 | -1.142 | 0.844 | -1.691 | 0.502 | -0.215 | -0.216 | -0.929 |
| hsa-miR-27b-3p | -0.031 | -0.183 | 0.049 | 0.822 | -0.068 | 0.173 | -0.246 | -1.379 | -2.005 | -2.267 | 0.192 | -0.070 | 0.691 |
| hsa-miR-28-3p | 0.012 | -0.643 | -0.077 | -1.472 | 0.261 | 0.743 | 0.034 | 0.708 | -0.899 | 0.684 | -0.868 | -0.126 | -0.710 |
| hsa-miR-28-5p | 0.002 | -0.256 | -0.987 | -0.806 | 0.317 | -0.696 | -0.088 | 0.259 | -1.971 | 0.624 | -0.703 | -0.236 | -1.734 |
| hsa-miR-30a-3p | 0.005 | -0.117 | -0.938 | -1.495 | 0.121 | -0.420 | -0.209 | -0.941 | 0.357 | -0.816 | -0.835 | -0.092 | 1.501 |
| hsa-miR-335-3p | -0.005 | -0.257 | -1.519 | -1.823 | -0.217 | -0.652 | -0.782 | -0.612 | 0.076 | 2.852 | -0.887 | -0.232 | -0.287 |
| hsa-miR-361-5p | 0.029 | -0.302 | 0.140 | 2.028 | 0.092 | 0.256 | 0.482 | -0.308 | -1.246 | -0.088 | 1.633 | 0.086 | -0.761 |
| hsa-miR-370-3p | -0.019 | -0.423 | -0.292 | -1.704 | -2.920 | 0.151 | 1.922 | 0.438 | -3.749 | 3.572 | -2.350 | 0.015 | 0.150 |
| hsa-miR-584-5p | 0.035 | -0.359 | 0.381 | -1.248 | -3.279 | 0.123 | 0.590 | 1.394 | -0.679 | 2.716 | 0.076 | -0.788 | 5.210 |
| hsa-miR-197-3p | 0.004 | -0.704 | 0.710 | 1.120 | -0.220 | -0.941 | 0.786 | 0.528 | 1.149 | 0.652 | 0.843 | -0.277 | -1.157 |

**Table S4**: The AUC statistical analysis.

| **ID** | **Marker** | **hsa.let.7d.5p** | **hsa.miR.142.3p** | **hsa.miR.199a.5p** | **hsa.miR.223.5p** | **hsa.miR.148b.3p** | **hsa.miR.340.5p** | **hsa.miR.421** |
| --- | --- | --- | --- | --- | --- | --- | --- | --- |
| **Colorectal cancer** | AUC | 0.9123 | 0.7961 | 0.76145 | 0.7492 | 0.9121 | 0.89405 | 0.95385 |
|  | SE.AUC | 0.01654 | 0.02621 | 0.02967 | 0.0303 | 0.01809 | 0.01904 | 0.01132 |
|  | LowerLimit | 0.87988 | 0.74473 | 0.70329 | 0.68982 | 0.87664 | 0.85673 | 0.93166 |
|  | UpperLimit | 0.94472 | 0.84747 | 0.81961 | 0.80858 | 0.94756 | 0.93137 | 0.97604 |
|  | p-value | <0.0001 | <0.0001 | <0.0001 | <0.0001 | <0.0001 | <0.0001 | <0.0001 |
| **Lung adenocarcinoma** | AUC | 0.93056 | 0.98611 | 0.90972 | 0.98264 | 0.86806 | 0.90278 | 0.95486 |
|  | SE.AUC | 0.04121 | 0.01345 | 0.0494 | 0.01562 | 0.06645 | 0.05259 | 0.03165 |
|  | LowerLimit | 0.84978 | 0.95976 | 0.8129 | 0.95203 | 0.73782 | 0.79971 | 0.89282 |
|  | UpperLimit | 1.01133 | 1.01246 | 1.00654 | 1.01325 | 0.99829 | 1.00584 | 1.0169 |
|  | p-value | <0.0001 | <0.0001 | <0.0001 | <0.0001 | <0.0001 | <0.0001 | <0.0001 |
| **Biliary tract cancer** | AUC | 0.93421 | 0.98026 | 0.99342 | 0.86842 | 0.92763 | 0.76316 | 0.98684 |
|  | SE.AUC | 0.05601 | 0.02247 | 0.0093 | 0.09945 | 0.05124 | 0.12005 | 0.01597 |
|  | LowerLimit | 0.82444 | 0.93622 | 0.97519 | 0.67349 | 0.8272 | 0.52786 | 0.95555 |
|  | UpperLimit | 1.04399 | 1.02431 | 1.01166 | 1.06335 | 1.02807 | 0.99845 | 1.01813 |
|  | p-value | <0.0001 | <0.0001 | <0.0001 | 0.00021 | <0.0001 | 0.02838 | <0.0001 |
| **Prostate cancer** | AUC | 0.93667 | 0.92933 | 0.74213 | 0.70987 | 0.79627 | 0.77547 | 0.79093 |
|  | SE.AUC | 0.02243 | 0.02444 | 0.03845 | 0.03956 | 0.03441 | 0.03676 | 0.03648 |
|  | LowerLimit | 0.8927 | 0.88142 | 0.66677 | 0.63233 | 0.72882 | 0.70342 | 0.71943 |
|  | UpperLimit | 0.98063 | 0.97724 | 0.8175 | 0.7874 | 0.86371 | 0.84751 | 0.86243 |
|  | p-value | <0.0001 | <0.0001 | <0.0001 | <0.0001 | <0.0001 | <0.0001 | <0.0001 |
| **Pancreatic cancer** | AUC | 0.90675 | 0.89484 | 0.95238 | 0.9127 | 0.9504 | 0.8869 | 0.99008 |
|  | SE.AUC | 0.04889 | 0.04997 | 0.02948 | 0.04364 | 0.03486 | 0.05379 | 0.00903 |
|  | LowerLimit | 0.81092 | 0.7969 | 0.89459 | 0.82716 | 0.88207 | 0.78148 | 0.97238 |
|  | UpperLimit | 1.00257 | 0.99278 | 1.01017 | 0.99823 | 1.01872 | 0.99233 | 1.00777 |
|  | p-value | <0.0001 | <0.0001 | <0.0001 | <0.0001 | <0.0001 | <0.0001 | <0.0001 |
| **Colon cancer** | AUC | 0.96753 | 0.71429 | 0.92857 | 0.88312 | 0.79221 | 0.87662 | 0.88312 |
|  | SE.AUC | 0.03464 | 0.10666 | 0.06044 | 0.0854 | 0.09935 | 0.07791 | 0.06677 |
|  | LowerLimit | 0.89963 | 0.50523 | 0.81011 | 0.71573 | 0.59748 | 0.72393 | 0.75225 |
|  | UpperLimit | 1.03543 | 0.92334 | 1.04703 | 1.05051 | 0.98694 | 1.02932 | 1.01399 |
|  | p-value | <0.0001 | 0.04454 | <0.0001 | 1.00E-05 | 0.00327 | <0.0001 | <0.0001 |
| **ALL** | AUC | 0.99777 | 0.98438 | 0.99554 | 0.93527 | 0.94866 | 0.92857 | 0.90179 |
|  | SE.AUC | 0.00316 | 0.01361 | 0.00541 | 0.03442 | 0.02975 | 0.03839 | 0.04919 |
|  | LowerLimit | 0.99158 | 0.95769 | 0.98494 | 0.86781 | 0.89035 | 0.85333 | 0.80538 |
|  | UpperLimit | 1.00395 | 1.01106 | 1.00613 | 1.00272 | 1.00697 | 1.00382 | 0.99819 |
|  | p-value | <0.0001 | <0.0001 | <0.0001 | <0.0001 | <0.0001 | <0.0001 | <0.0001 |
| **AML** | AUC | 0.86875 | 0.75764 | 0.92292 | 0.83472 | 0.92222 | 0.74653 | 0.98125 |
|  | SE.AUC | 0.04443 | 0.05544 | 0.02866 | 0.04569 | 0.02916 | 0.05912 | 0.01071 |
|  | LowerLimit | 0.78168 | 0.64897 | 0.86673 | 0.74518 | 0.86506 | 0.63066 | 0.96026 |
|  | UpperLimit | 0.95582 | 0.86631 | 0.9791 | 0.92426 | 0.97938 | 0.86239 | 1.00224 |
|  | p-value | <0.0001 | <0.0001 | <0.0001 | <0.0001 | <0.0001 | 3.00E-05 | <0.0001 |
| **Nasopharyngeal** | AUC | 0.97222 | 0.82639 | 0.93056 | 0.8588 | 0.83102 | 0.7662 | 0.70139 |
|  | SE.AUC | 0.01901 | 0.06521 | 0.03811 | 0.06545 | 0.06558 | 0.08652 | 0.08749 |
|  | LowerLimit | 0.93496 | 0.69857 | 0.85586 | 0.73051 | 0.70248 | 0.59662 | 0.52991 |
|  | UpperLimit | 1.00948 | 0.9542 | 1.00525 | 0.98708 | 0.95956 | 0.93579 | 0.87287 |
|  | p-value | <0.0001 | <0.0001 | <0.0001 | <0.0001 | <0.0001 | 0.00209 | 0.02134 |

**Table S5:** table of 26 best-fitted target genes.

| Gene symbol | Degree | Betweenness |
| --- | --- | --- |
| 1. CCND1 | 3 | 4.888214 |
| 1. IGF1R | 3 | 4.888214 |
| 1. LBR | 3 | 5.398159 |
| 1. SOD2 | 3 | 5.398159 |
| 1. TGFBR1 | 3 | 6.852381 |
| 1. WASL | 3 | 5.037355 |
| 1. ZNF264 | 3 | 4.202315 |
| 1. AGO1 | 3 | 5.653285 |
| 1. TMTC3 | 3 | 4.887118 |
| 1. MFSD8 | 3 | 4.202315 |
| 1. MTX3 | 3 | 5.262162 |
| 1. LDLR | 3 | 6.753139 |
| 1. MECP2 | 3 | 6.659205 |
| 1. SLC38A2 | 3 | 6.659205 |
| 1. PANK3 | 3 | 6.438247 |
| 1. ARHGAP12 | 3 | 6.438247 |
| 1. HOXA9 | 3 | 5.664145 |
| 1. MKLN1 | 3 | 4.262527 |
| 1. MORF4L1 | 3 | 4.662166 |
| 1. SLC7A11 | 4 | 9.49496 |
| 1. KLHL15 | 3 | 4.262527 |
| 1. MCFD2 | 3 | 6.093646 |
| 1. KLF6 | 3 | 4.80921 |
| 1. JARID2 | 3 | 5.346734 |
| 1. ID4 | 3 | 5.393181 |
| 1. CREBRF | 3 | 5.393181 |

**Table S6**: Statistical analysis of three datasets used for validation in whole blood. The mean of expressions was used in each dataset.

| **miRNA** | **E-MTAB-8026** | | | **GSE118613** | | **GSE40738** | |
| --- | --- | --- | --- | --- | --- | --- | --- |
|  | Healthy (N=964) | Lung Carcinoma (N=606) | Lung Disease (N=593) | Healthy (N=30) | Nasopharyngeal Carcinoma (N=120) | Healthy (N=58) | Lung Cancer (N=119) |
| miR-142-3p | 1.610 | 4.783 | -1.034 | 3.339 | -1.873 | 0.1137 | 1.969 |
| P value | Control | <0.0001 | <0.0001 | Control | <0.0001 | Control | <0.0001 |
| miR-199a-5p | 2.704 | 1.641 | 5.085 | 1.652 | 2.522 | 1.510 | 3.514 |
| Pvalues | Control | <0.0001 | <0.0001 | Control | 0.0015 | Control | <0.0001 |
| miR-223-5p | 3.209 | 3.587 | 2.976 | -2.409 | 0.6354 | -1.162 | 0.8681 |
| Pvalues | Control | <0.0001 | 0.0002 | Control | <0.0001 | Control | <0.0001 |
| let-7d-5p | 1.669 | 4.914 | 0.4135 | -0.4509 | 2.599 | -0.7995 | 2.779 |
| Pvalues | Control | <0.0001 | <0.0001 | Control | <0.0001 | Control | <0.0001 |
| miR-148b-3p | 7.272 | 7.879 | 4.524 | 2.158 | -0.05848 | 1.356 | 2.879 |
| Pvalues | Control | <0.0001 | <0.0001 | Control | <0.0001 | Control | <0.0001 |
| miR-340-5p | 1.501 | 4.519 | 0.6445 | -3.829 | 1.492 | -1.308 | 0.7828 |
| Pvalues | Control | <0.0001 | <0.0001 | Control | <0.0001 | Control | <0.0001 |
| miR-421 | 1.551 | 4.508 | -1.994 | -1.601 | 1.367 | -1.286 | 1.334 |
| Pvalues | Control | <0.0001 | <0.0001 | Control | <0.0001 | Control | <0.0001 |

Table S7: Table of clinical information of patient and control group that was used as validation. There were no clinical data on smocking history on data GSE118613 (Fehlmann et al., 2020, Wen, 2018, Patnaik et al., 2012).

| **Studies** | | **E-MTAB-8026** | | | **GSE118613** | | | **GSE40738** | |
| --- | --- | --- | --- | --- | --- | --- | --- | --- | --- |
| **Diagnosis (Sample Size)** | | **Healthy (N=964)** | **Lung Carcinoma (N=606)** | **Lung Disease (N=593)** | **Healthy (N=30)** | **Nasopharyngeal Carcinoma (N=120)** | | **Healthy (N=58)** | **Lung Cancer (N=119)** |
| **Age(mean)** | | 52.4 | 65.4 | 65.8 | 42.9 | | 55.9 | 61.1 | 63.5 |
| **Sex (%)** | **Male** | 51.7 | 67.1 | 65.5 | 24 | | 91 | 49 | 51 |
|  | **Female** | 48.3 | 32.9 | 34.5 | 6 | | 29 | 51 | 49 |
| **Smocking (%)** | **Never** | 4.8 | 9.2 | 64.4 | - | | - | - | - |
|  | **Past** | 71.4 | 33.3 | 30.0 | - | | - | 99 | 93 |
|  | **Present** | 23.8 | 57.5 | 5.6 | - | | - | - | - |

**
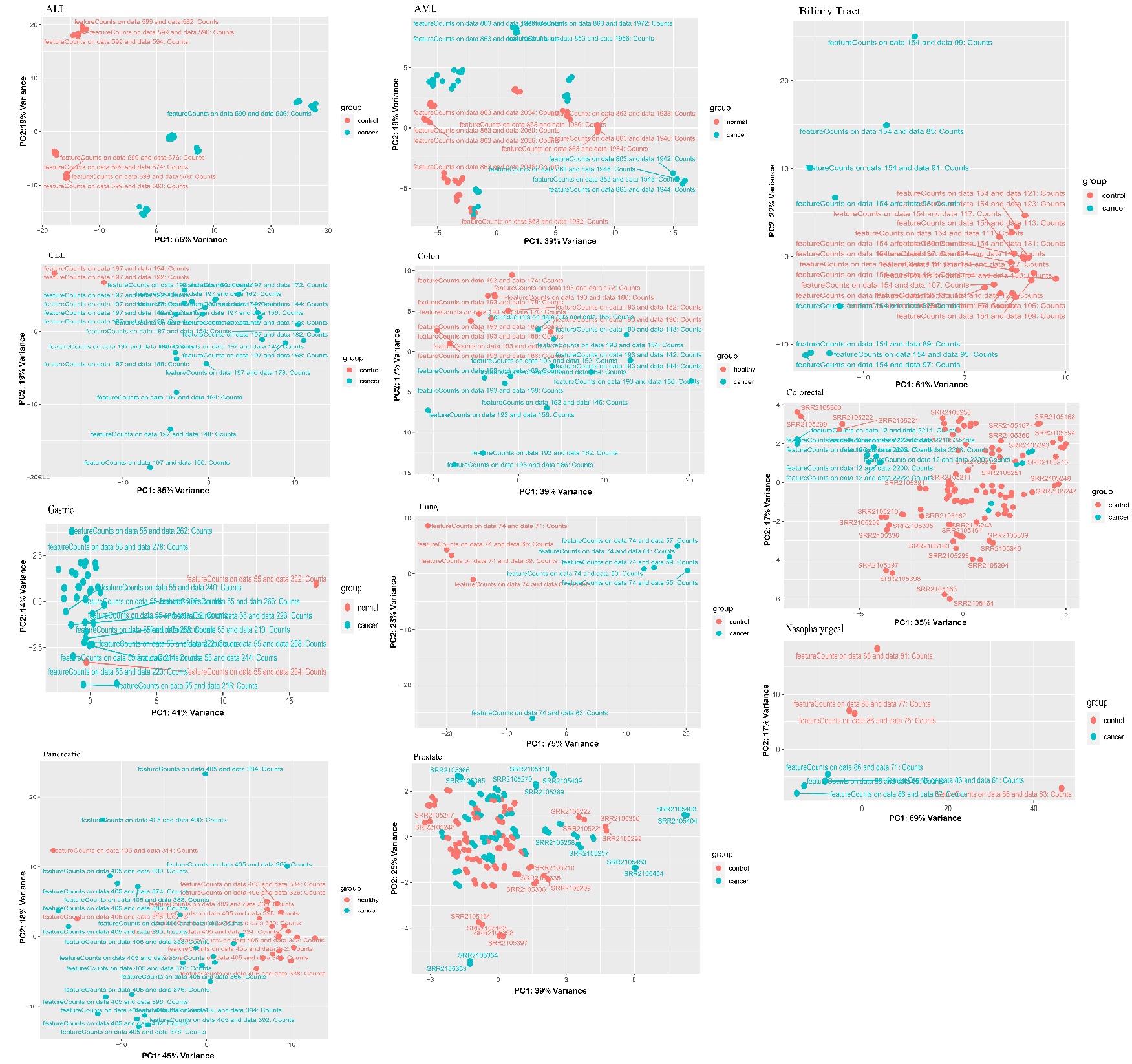
**

**Figure S1: PCA lot for each cancer type in compare to their specific control samples.**

FEHLMANN, T., KAHRAMAN, M., LUDWIG, N., BACKES, C., GALATA, V., KELLER, V., GEFFERS, L., MERCALDO, N., HORNUNG, D. & WEIS, T. 2020. Evaluating the use of circulating microRNA profiles for lung cancer detection in symptomatic patients. *JAMA oncology,* 6**,** 714-723.

MUSSACK, V., WITTMANN, G. & PFAFFL, M. W. 2021. On the trail of blood doping—microRNA fingerprints to monitor autologous blood transfusions in vivo. *American Journal of Hematology,* 96**,** 338-353.

PATNAIK, S. K., YENDAMURI, S., KANNISTO, E., KUCHARCZUK, J. C., SINGHAL, S. & VACHANI, A. 2012. MicroRNA expression profiles of whole blood in lung adenocarcinoma.

WEN, W. 2018. Two miRNA signatures for identifying nasopharyngeal carcinoma from head-neck tumors and the normal. *Annals of Oncology,* 29**,** ix99-ix100.
